# Ring Augmentation of the Roux-en-Y Gastric Bypass: A Propensity Score Matched Analysis of 5-Year Follow-Up Results

**DOI:** 10.1007/s11695-025-07706-x

**Published:** 2025-01-30

**Authors:** Marijn T. F. Jense, Floris F. E. Bruinsma, Simon W. Nienhuijs, Ronald S. L. Liem, Perla J. Marang-van de Mheen, Jan Willem M. Greve, Evert-Jan G. Boerma

**Affiliations:** 1 Zuyderland Medisch Centrum, Sittard, Netherlands; 2 Maastricht University, Maastricht, Netherlands; 3 Dutch Institute for Clinical Auditing, Leiden, Netherlands; 4 Catharina Ziekenhuis, Eindhoven, Netherlands; 5 Groene Hart Ziekenhuis, Gouda, Netherlands; 6 Nederlandse Obesitas Kliniek, Zeist, Netherlands; 7 Delft University of Technology, Delft, Netherlands

**Keywords:** Ring augmented gastric bypass, Ring augmented Roux-en-Y gastric bypass, Banded bypass, Ring augmentation, Recurrent weight gain, Real-world data

## Abstract

**Background:**

The ring-augmented Roux-en-Y gastric bypass (raRYGB) has been reported to result in higher long-term weight loss compared to regular Roux-en-Y gastric bypass (RYGB). However, the type of ring used varied within studies, leading to heterogeneity in reported results. Therefore, this study compares the 5-year results of RYGB with and without ring augmentation using a specific prefabricated gastric ring.

**Methods:**

All consecutive patients from a single center who received primary raRYGB between June 1, 2016, and May 31, 2018, with complete 5-year follow-up data were included and compared to a propensity score matched cohort receiving RYGB in the same period from the Dutch Audit for Treatment of Obesity. To ensure fair effect estimation of placing a ring, only RYGB procedures with alimentary and biliopancreatic limb lengths similar to those of the raRYGB were considered eligible. The primary outcome was percentage total weight loss (%TWL). Secondary outcomes included recurrent weight gain (RWG), obesity complication improvement, and complications.

**Results:**

In total, 592 matched patients were analyzed. raRYGB was associated with higher %TWL at 5 years (31.5% versus 28.0%, *β* = 3.59, 95% CI [2.09–5.09], *p* < 0.01) and lower odds on RWG (odds ratio = 0.56, 95% CI [0.38–0.83], *p* < 0.01). Improvement of obesity complications and short-term complication rates were comparable in both groups. After 5 years, 13 patients (4%) had experienced ring-related complications needing reoperation.

**Conclusions:**

At 5 years, raRYGB was associated with higher %TWL and lower odds on RWG. The occurrence of ring-related complications was limited.

**Supplementary Information:**

The online version contains supplementary material available at 10.1007/s11695-025-07706-x.

## Introduction

Different bariatric procedures are practiced worldwide, with sleeve gastrectomy (SG) and Roux-en-Y gastric bypass (RYGB) being the most commonly performed surgeries [1]. Compared with SG, the RYGB is known to have better results in terms of long-term weight loss as SG comes with an increased risk of recurrent weight gain (RWG) [2, 3]. However, still 10–35% of patients receiving RYGB experience suboptimal clinical response or RWG, potentially leading to secondary surgery [3–7]. Since complication risks after revision or conversion RYGB are up to three times higher compared with primary RYGB, the need to prevent secondary surgery is an important consideration when performing metabolic bariatric surgery (MBS) [8, 9].

Previous studies have shown that placing a ring around the gastric pouch, known as ring-augmented Roux-en-Y gastric bypass (raRYGB) [10]—previously called banded RYGB—leads to greater weight loss in the medium to long term [11–13]. This effect could be either due to better initial weight loss or reduced RWG over time [12]. Although multiple studies have investigated the effects of raRYGB, many utilized self-fabricated rings made from materials such as bovine patches, polypropylene mesh, or ventricular drains, which were not originally designed to be placed around a gastric pouch [13–16]. This may have contributed to the relatively high rates of reported food intolerance and inconsistent weight loss results.

To date, only one cohort study has directly compared outcomes of RYGB with raRYGB using a ring specifically designed for placement around a gastric pouch [17], reporting greater long-term weight loss and less RWG in the raRYGB cohort. However, considerable baseline differences seemed to be present between both treatment groups with heavier patients in the raRYGB group, which was not appropriately tested nor adjusted in subsequent analyses. Moreover, the study did not report weight loss as a percentage of total weight loss (%TWL), which is now considered the standard metric for fair comparison [18, 19]. In addition, as it was a non-randomized study, there may be an underlying reason why some patients received RYGB instead of raRYGB. Where randomization deals with such confounding by indication, techniques such as propensity score matching can be used for observational data to result in reliable estimates of the treatment effect [20]. These issues highlight the need for additional research with population-based data to circumvent the problem of selected patients participating in randomized trials.

Therefore, the aim of the current study was to compare the 5-year results of the RYGB with and without ring augmentation using a prefabricated gastric ring, applying propensity score matching to create balanced groups with population-based data. Because intestinal limb length can affect outcomes [21–26], only procedures (i.e., RYGB and raRYGB) with similar biliopancreatic (BPL) and alimentary limb (AL) lengths were included to ensure fair comparison and to estimate the isolated effect of adding a ring.

## Methods

### Data Collection

Data were derived from the Dutch Audit for Treatment of Obesity (DATO), which is a mandatory national quality registry for MBS [27, 28]. All Dutch bariatric centers participate in this registry, thereby delivering valuable real-world insights into outcomes after MBS. DATO collects patient and procedure characteristics, complication rates, and yearly follow-up data on weight and obesity complications up to 5 years after surgery. Data verification has proven the high validity of the data [29]. Hospitals have registration of postoperative complications embedded in routine postoperative care until 30 days after discharge, so that long-term complications may be underreported in DATO. Given that ring-related complications are an important consideration in deciding which procedure to perform, the electronic patient files (EPF) of the hospital performing raRYGB were consulted to ensure a more specific evaluation of any ring-related complications up to 5 years after surgery. The study was presented to the medical ethics committee METC Z who waived the need for ethical approval under Dutch law.

### Procedures

A silicone ring specifically designed to be added to bariatric procedures, the MiniMIZER™ gastric ring, was used in all raRYGB cases [30]. This flexible but sturdy ring is loosely placed around the newly created pouch so that it will not cause any restriction. Its placement is estimated to require approximately 3 to 5 min. Depending on the pouch size, the circumference of the ring can be adjusted from 6.5 to 8 cm. In the current study, the pouch was calibrated around a 40 Charrière gastric tube in all cases.

### Patient Selection

All patients receiving primary raRYGB in Zuyderland Medical Center (ZMC) between June 1, 2016, and May 31, 2018, were included and considered the treatment group. Patients receiving RYGB in the same period in any Dutch hospital including ZMC were considered candidates for the reference group. As intestinal limb lengths may affect outcomes, the lengths of the BPL and AL needed to be similar between both treatment groups [21–25]. Additional data collection in the EPF confirmed that all raRYGB cases were performed with BPL lengths of 60 cm and AL lengths of 120 cm. Therefore, only primary RYGB with BPL lengths of 50–70 cm and AL lengths of 100–150 cm were considered eligible for inclusion in the reference group. Patients were excluded if they had missing baseline characteristics (i.e., age, sex, body mass index (BMI, kg/m^2^), American Society of Anesthesiologists score (ASA-score)), procedure characteristics (i.e., BPL and AL lengths), obesity complication status (i.e., diabetes mellitus (DM), hypertension, dyslipidemia, obstructive sleep apnea syndrome (OSAS), gastroesophageal reflux disease (GERD), and musculoskeletal pain), if they did not meet the criteria for MBS according to the national guidelines [31] or if they had less than 5 years of follow-up data available.

### Outcomes

The primary outcome was %TWL (calculated as [baseline weight − follow-up weight]/baseline weight × 100) at 5 years after surgery, both on a continuous scale and dichotomized as achieving at least 25%TWL. Secondary outcomes included two weight-related adverse outcomes: excessive weight loss (i.e., dropping below a BMI of 20 kg/m^2^ at any time point) and RWG using the standard definition from the International Federation for the Surgery of Obesity and Metabolic Disorders (IFSO) being weight gain of > 30% from the initial weight loss [32]. Patients experiencing RWG at any time-point up to 5 years were considered to have experienced RWG, regardless of their actual weight loss status at 5 years.

Other secondary outcomes were severe postoperative complication rates (defined as Clavien-Dindo grade ≥ 3 occurring within 30 days), ring-related complications (i.e., any complication such as erosion, dislocation, or dysphagia) up to 5 years after surgery, prolonged length of the initial hospitalization (defined as ≥ 3 days), readmission within 30 days after surgery, and improvement of the aforementioned obesity complications at 5 years after surgery. Improvement is defined as either partial or complete remission or relief of symptoms of the obesity complication in question. Exact definitions and criteria for obesity complication improvement have been described previously [33].

### Statistical Analysis

Differences in baseline characteristics between both treatment groups were assessed. To create balanced groups of similar patients, 1:1 propensity score matching was performed using the nearest neighbor method with a caliper width of 0.2, based on the logit of the propensity score [34, 35]. All available baseline characteristics mentioned before were used for calculating the propensity scores. A standardized mean difference (SMD) of < 0.1 was considered to indicate balanced groups [20, 36].

After matching, both treatment groups were compared on outcomes, using linear regression analysis for continuous outcomes and logistic regression analysis for dichotomous outcomes, including the treatment group and the propensity score as independent variables. In case of an imbalance in any of the baseline characteristics (i.e., SMD ≥ 0.10), this variable was included in the regression analysis to correct for this imbalance. Improvement of obesity complications was assessed within the patient group having the specific obesity complication at baseline. An alpha of < 0.05 was considered statistically significant for all analyses. Statistical analyses were performed using RStudio version 2023.06.1 (R Foundation for Statistical Computing, Vienna, Austria), and matching was done using the MatchIt package (version 4.5.5).

### Exploratory Analysis

Most patients have a long-lasting response to MBS; however, some experience significant RWG. If we hypothesize that 80% of patients have a good and durable response to MBS (and therefore only experience some RWG, e.g., a few kilograms), that means that 20% of patients would be susceptible to more serious RWG (in line with the 10–35% as reported previously [3–7]). Therefore, boxplots were created within both matched treatment groups displaying the 80% of patients experiencing the least RWG (i.e., some RWG or no RWG at all) and the 20% of patients experiencing the highest RWG. This enabled us to compare the amount of RWG in the 20% susceptible to more serious RWG between both procedures. The weight loss trajectories for both procedures were assessed in a line graph for all matched patients by plotting the average weight loss at each follow-up year.

## Results

A total of 507 patients with complete baseline characteristics underwent raRYGB during the specified period. Additionally, 4514 patients who received RYGB with similar intestinal limb lengths were identified. Among these cohorts, 301 and 1227 patients, respectively, had 5-year follow-up data available. Patients receiving RYGB were operated in 11 different hospitals, including the hospital performing raRYGB. Matching resulted in 296 matched patients per treatment group with balanced baseline characteristics, as shown in Table 1. The median BPL length in the RYGB group was 60 cm (inter-quartile range (IQR) [50–70]), and the median AL length was 150 cm (IQR [150–150]), both before and after matching. In the raRYGB group, all patients had BPL lengths of 60 cm and AL lengths of 120 cm. All rings in the raRYGB group were closed at a circumference of 6.5–7.5 cm.

**Table 1:** Baseline characteristics before and after matching

	*Before matching*			*After matching*		
	**RYGB**	**raRYGB**	SMD	**RYGB**	**raRYGB**	SMD
*n*	1227	301		296	296	
*Age (median [range])*	47 [19, 68]	48 [19, 67]	0.06	47 [19, 65]	47 [19, 67]	0.07
*Bmi (median [range])*	41.3 [34.0, 64.8]	41.7 [34.4, 60.5]	0.11	41.5 [34.7, 62.0]	41.8 [34.4, 60.5]	0.04
*Sex (n, %)*						
*Male*	209 (17.0)	73 (24.3)	0.18	70 (23.6)	70 (23.6)	0.00
*Female*	1018 (83.0)	228 (75.7)		226 (76.4)	226 (76.4)	
*ASA-score (n, %)*						
< *3*	791 (64.5)	162 (53.8)	0.22	160 (54.1)	161 (54.4)	0.01
≥ *3*	436 (35.5)	139 (46.2)		136 (45.9)	135 (45.6)	
*Diabetes mellitus (n, %)*						
*Not present*	1016 (82.7)	237 (78.7)	0.31	240 (81.1)	237 (80.1)	0.05
*Present without medication*	38 (3.1)	32 (10.6)		23 (7.8)	27 (9.1)	
*Present with medication*	173 (14.2)	32 (10.6)		33 (11.1)	32 (10.8)	
*Hypertension (n, %)*						
*Not present*	804 (65.5)	185 (61.5)	0.08	196 (66.2)	185 (62.5)	0.08
Present*	423 (34.5)	116 (38.5)		100 (33.8)	111 (37.5)	
*Dyslipidemia (n, %)*						
*Not present*	992 (80.8)	239 (79.4)	0.27	235 (79.4)	237 (80.1)	0.08
*Present without medication*	81 (6.7)	40 (13.3)		33 (11.1)	37 (12.5)	
*Present with medication*	154 (12.5)	22 (7.3)		28 (9.5)	22 (7.4)	
*OSAS (n, %)*						
*Not present*	976 (79.5)	248 (82.4)	0.53	246 (83.1)	244 (82.4)	0.02
*Present without therapy*	111 (9.0)	52 (17.3)		49 (16.6)	51 (17.2)	
*Present with therapy*	140 (11.5)	1 (0.3)		1 (0.3)	1 (0.3)	
*GERD (n, %)*						
*Not present*	1048 (85.4)	253 (84.1)	0.22	248 (83.8)	248 (83.8)	0.03
*Present without medication*	60 (4.9)	5 (1.7)		4 (1.4)	5 (1.7)	
*Present with medication*	119 (9.7)	43 (14.3)		44 (14.9)	43 (14.5)	
*Musculoskeletal pain (n, %)*						
*Not present*	729 (59.4)	109 (36.2)	0.50	106 (35.8)	109 (36.8)	0.02
Present**	498 (40.6)	192 (63.8)		190 (64.2)	187 (63.2)	
*Limb lengths (median [range])*						
*Biliopancreatic limb*	60 [50, 70]	60 [60, 60]		60 [50, 70]	60 [60, 60]	
*Alimentary limb*	150 [100, 150]	120 [120, 120]		150 [100, 150]	120 [120, 120]	

*RYGB* regular Roux-en-Y gastric bypass, *raRYGB* ring augmented Roux-en-Y gastric bypass, *SMD* standardized mean difference, *n* number of patients, *ASA* American Society of Anaesthesiologists, *OSAS* obstructive sleep apnea syndrome, *GERD* gastroesophageal reflux disease.

*Hypertension with and without medication were combined to improve balance.

**The available data did not discriminate between musculoskeletal pain with or without medication.

### Weight Loss

The mean %TWL at 5 years was 28.0% for RYGB and 31.5% for raRYGB (*β* = 3.59, 95% CI [2.09–5.09], *p* < 0.01), with patients receiving raRYGB having higher odds to achieve ≥ 25% TWL (odds ratio (OR) 2.45, 95% confidence interval (CI) [1.70–3.52], *p* < 0.01, Table 2). In addition, patients receiving raRYGB had lower odds to experience RWG (OR 0.62, 95% CI [0.42–0.90], *p* = 0.01, Table 2). Excessive weight loss occurred in seven patients receiving raRYGB and four patients receiving RYGB which was not statistically different. A table presenting additional weight-related outcomes, such as delta BMI and percentage excess weight loss (%EWL), has been included in the supplementary files to enable comparison with existing data (supplementary Table 1).

**Table 2:** Five-year outcomes of ring augmented versus standard Roux-en-Y gastric bypass in matched patients

	***RYGB***	***raRYGB***	***OR (95% CI)***	***p-value***
	**(*n* = 296)**	**(*n* = 296)**		
*Weight-related outcomes*				
≥ *25%TWL (n, %)*	180 (60.8)	234 (79.1)	2.45 (1.70–3.52)	< 0.01
*Recurrent weight gain (n, %)*	86 (29.1)	60 (18.6)	0.62 (0.42–0.90)	0.01
*Excessive weight loss (n, %)*	4 (1.4)	7 (2.4)	1.77 (0.51–6.11)	0.36
*Complication-related outcomes*				
*Hospital stay* ≥ *3 days (n, %)*	21 (7.1)	5 (1.7)	0.22 (0.08–0.60)	< 0.01
*Readmission* < *30 days (n, %)*	9 (3.0)	15 (5.1)	1.70 (0.73–3.94)	0.22
*CD3* + < *30 days (n, %)*	6 (2.0)	10 (3.4)	1.67 (0.60–4.67)	0.33
*Obesity complication improvement*				
*Diabetes mellitus (%)*	85.7	78.7	0.62 (0.21–1.79)	0.37
*Hypertension (%)*	80.9	66.3	0.47 (0.23–0.93)	0.03
*Dyslipidemia (%)*	72.1	56.3	0.48 (0.20–1.16)	0.10
*OSAS (%)*	94.4	95.1	1.29 (0.16–10.1)	0.81
*GERD (%)*	95.2	86.7	0.24 (0.02–3.46)	0.29
*Musculoskeletal pain (%)*	70.1	79.2	1.62 (0.91–2.90)	0.10

Reference = RYGB.

Recurrent weight gain = recurrent weight gain of > 30% from initial weight loss.

Excessive weight loss = resulting in BMI < 20 kg/m.^2^

*RYGB* regular Roux-en-Y gastric bypass, *raRYGB* ring augmented Roux-en-Y gastric bypass, *n* number of patients, *OR* odds ratio, *CI* confidence interval, *%TWL* percentage total weight loss, *BMI* body mass index, *CD* Clavien-Dindo, *n* number of patients with the obesity complication at baseline, *OSAS* obstructive sleep apnea syndrome, *GERD* gastro-esophageal reflux disease.

### Obesity Complications and Complications

Availability of obesity complication status at follow-up was evenly distributed between both procedures (see supplementary Table 2). There were no significant differences in improvement of obesity complications at 5 years, except for raRYGB having lower odds to result in improvement of hypertension (OR 0.47, 95% CI [0.23–0,93], Table 2). Patients who underwent raRYGB had lower odds to experience prolonged length of stay (odds ratio (OR) 0.47, 95% CI (0.23–0.93), *p* < 0.01). No statistically significant differences were found regarding postoperative complications and readmission rates (Table 2).

### Ring-Related Complications

In the raRYGB group, 13 (4.4%) ring-related complications were reported, all leading to reoperation. The median time between primary surgery and reoperation was 32 months (range 6–56). In seven (2.4%) patients, the ring was removed; in all others, the ring was replaced with a wider circumference compared to the initial placement. Dysphagia was the most common complication among the ring-related complications (8/13; see Table 3). Of the patients with dysphagia, four had a ring size of 6.5 cm, three had 7 cm, and one had 7.5 cm. After reoperation, the ring-related complaints were resolved in all patients. No erosions of the ring were seen in this study group.

**Table 3:** Overview of ring-related complications

	*Replaced*	*Removed*	*Total*
*Dislocation*		1	1
*Dysphagia*	5	3	8
*Abdominal complaints*		2	2
*Stenosis*		1	1
*Herniation through ring*	1		1
*Total*	6	7	13

## Exploratory Analysis

**Fig. 1:**
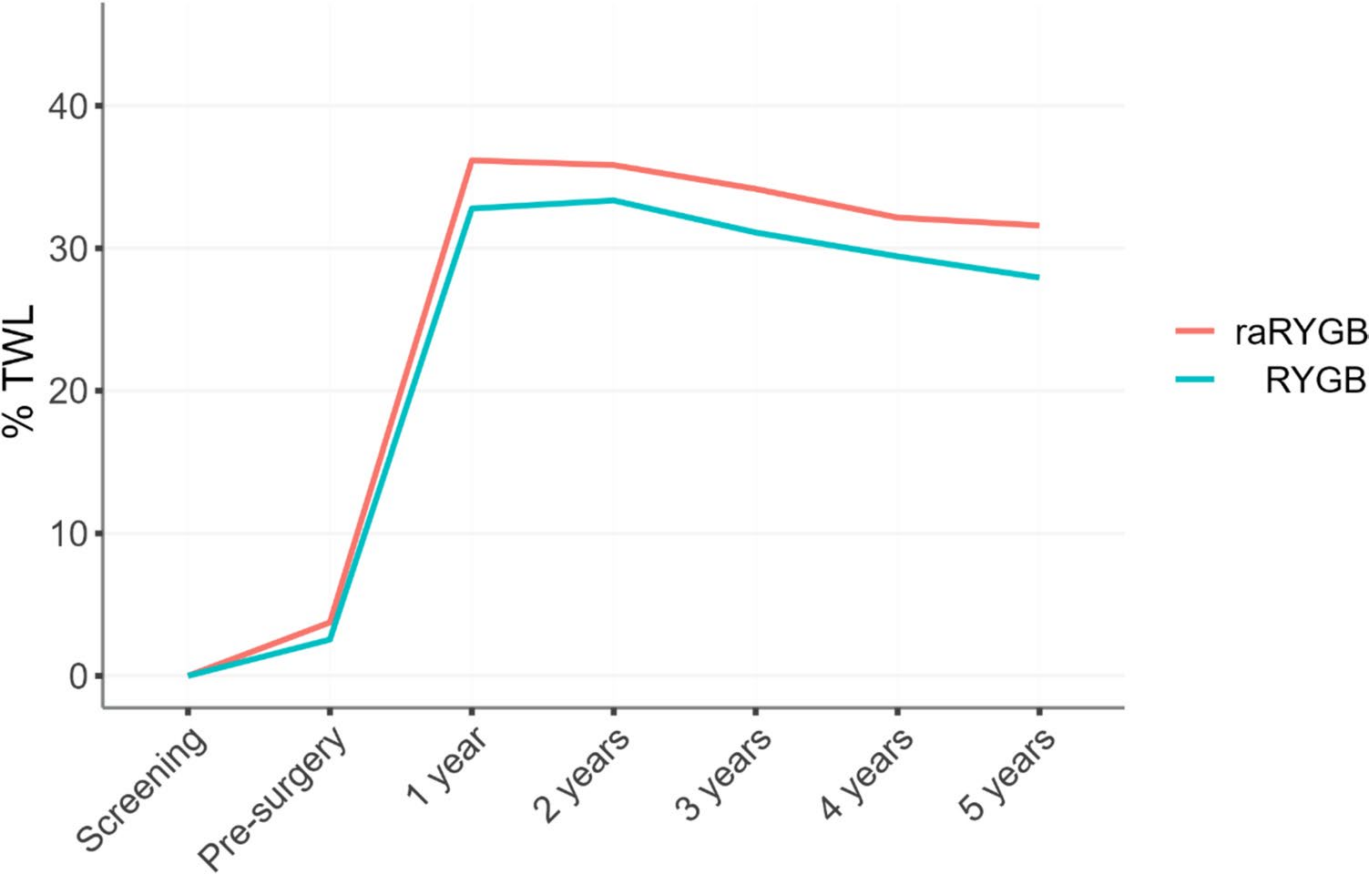
Weight loss trajectories for raRYGB and RYGB. The available follow-up weights between 1 and 5 years were taken to calculate the mean for each time point, meaning that these might be based on fewer patients than are available for the 5-year follow-up moment if patients missed intermediate consultations at the outpatient clinic. %TWL, percentage total weight loss; raRYGB, ring-augmented Roux-en-Y gastric bypass; RYGB, regular Roux-en-Y gastric bypass

**Fig. 2:**
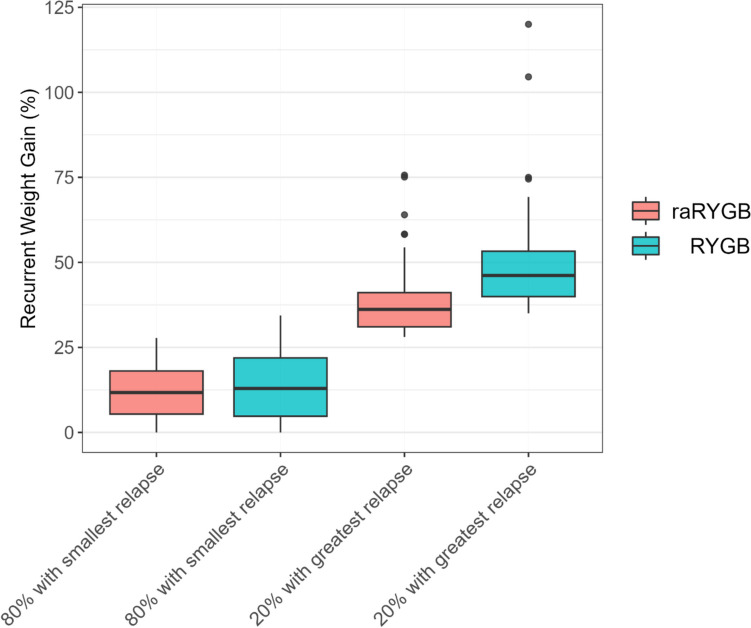
Boxplots showing the recurrent weight gain for patients receiving raRYGB and RYGB, subdivided into groups based on whether patients have experienced relatively much recurrent weight gain or not. 80% with smallest relapse = the 80% of patients who experienced the least recurrent weight gain within the procedure in question (i.e., RYGB or raRYGB); 20% with greatest relapse = the 20% of patients who experienced the most recurrent weight gain within the procedure in question (i.e., RYGB or raRYGB); raRYGB, ring-augmented Roux-en-Y gastric bypass; RYGB, regular Roux-en-Y gastric bypass

Figure 1 shows the weight loss trajectory over 5 years for patients receiving raRYGB versus RYGB. raRYGB resulted in more %TWL at each point of follow-up, but these lines do not diverge over time. When focusing on the 20% of patients with the highest RWG in each treatment group, Fig. 2 shows that the amount of RWG is lower for patients receiving raRYGB while the boxplots for the 80% with the least RWG appear fairly similar.

## Discussion

At 5 years after surgery, raRYGB resulted in significantly higher weight loss, higher odds to achieve ≥ 25% TWL, and lower odds on RWG. raRYGB had lower odds to improve hypertension but no differences in any of the other obesity complications were found. Performing raRYGB did not have higher rates of short-term complications, readmissions, or prolonged hospital stays and was not associated with a higher chance to experience excessive weight loss. The exploratory analysis showed that for the 20% of patients with the highest RWG within both treatment groups, the amount of RWG was lower among patients with ring augmentation.

### Weight Loss

The superior weight loss of the raRYGB as found in the current study has been described before, mainly in studies with a ring not specifically designed for this purpose [13, 14, 37, 38]. The strongest evidence available comes from a meta-analysis of three randomized controlled trials [11], in which the raRYGB was found to result in about 5% extra excess weight loss at 2–5 years. To our knowledge, the present study is the first to use real-world data from a national quality registry, thereby including a more representative and larger patient population than the selection of patients participating in randomized trials. The current study assessed almost 600 patients with 5-year follow-up data, where the meta-analysis mostly included patients with shorter follow-up data and only 43 patients who completed the 5-year follow-up. This study thereby adds more reliable evidence with respect to longer-term follow-up. Furthermore, the meta-analysis included results from studies using different types of self-fabricated rings which will likely introduce heterogeneity in the estimated effect, where the current study provided a more specific estimate associated with the use of the MiniMIZER ring, a ring specifically designed to be placed around a gastric pouch.

Considering RWG, there is only very limited literature available describing this association [17] and the current study contributes by finding lower odds of RWG associated with raRYGB. As shown by the exploratory analysis, the 80% of patients with the least RWG within both treatment groups, had similar amounts of RWG. This suggests that patients who react well to MBS might achieve a little more %TWL when raRYGB is performed with similar amounts of RWG for up to 5 years. However, for the 20% of patients with the highest RWG, the ring augmentation may be most beneficial, given the lower amount of RWG, which might reduce the need for additional treatment. Unfortunately, one does not know in advance which patients will experience more or less RWG, and therefore some patients will be put at risk for ring-related complications while their benefit will only be limited to some additional weight loss. However, as ring-related complications only occurred in 4.4% of patients, this risk appears fairly low.

There is a paucity in literature on why ring augmentation might prevent RWG. While numerous factors including genetics, diet, and psychiatric conditions have been associated with RWG [39], it is unclear which impact ring augmentation of the pouch has on the physiology behind weight loss maintenance. As the ring is placed loosely around the pouch, it is likely not related to restriction. It has been hypothesized that raRYGB results in increased satiety and may prevent pouch dilatation [40]. As delayed gastric emptying has been found to be associated with increased satiety and weight loss, this might play a role, but additional research is needed to test this hypothesis [41, 42].

### Ring-Related Complications

One of the major concerns when performing raRYGB is the additional complication risk. In the current study, 13 patients (4.4%) experienced complications directly related to the placement of the ring, consistent with rates reported in other studies [12, 14, 17]. The meta-analysis by Shoar et al., however, reported a high incidence of food intolerance (29%) [11]. This could be explained by the circumference of the placed rings in those studies, being 5.5–6.5 cm, which may result in impaired passage of food. In the current study, only circumferences of 6.5–7.5 cm were used, thereby preventing passage complaints in most cases. Nevertheless, eight patients (2.7%) experienced dysphagia, likely due to the smaller ring circumference used in these patients, with four having circumferences of 6.5 cm. Following these findings, the hospital performing raRYGB discontinued the use of circumferences smaller than 7 cm, resulting in reduced dysphagia rates afterwards.

The additional complication risk should be interpreted in light of the risk for secondary surgery. Since more patients experienced RWG in the RYGB group, these patients would have a higher probability to need secondary bariatric surgery for suboptimal initial clinical response at some point, which is one of the leading indications for revisional surgery [43–46]. Although obesity management medications are increasingly prescribed for patients experiencing RWG, these medications must be used life-long and are often not reimbursed [6, 47–50]. Therefore, it is likely that surgery will remain one of the cornerstones in the treatment of RWG in the future. Furthermore, secondary surgery comes at a higher complication risk, potentially leading to reoperation [8, 9, 51, 52]. All taken together, performing raRYGB could reduce the chance of additional surgery. As RWG is difficult to treat and ring-related complications were limited, augmentation of the RYGB with a non-adjustable silicone ring such as the MiniMIZER ring, should be seriously considered for standard practice.

### Obesity Complication Improvement

Except for improvement in hypertension, no statistically significant differences in obesity complication reduction were found between both treatments. It is unclear how the lower odds to improve hypertension associated with ring augmentation should be explained, but that can be the focus of future research.

### Limitations and Strengths

Some limitations of the current study should be noted. First, propensity score matching is not able to adjust for unmeasured confounders such as peri-operative guidance and socioeconomic status [20]. The implicit assumption therefore is that these unmeasured confounders become evenly distributed between both treatment groups by the pseudo-randomization of propensity score matching. Second, the current study was unable to directly compare long-term complications, an important concern when comparing both procedures. Although additional EPF research was performed in the hospital performing raRYGB, thereby providing valuable data on ring-related complications, it was not feasible to assess long-term complications across all other hospitals within the RYGB group. Third, follow-up numbers of obesity complications were high for DM and hypertension but relatively low for GERD. However, as the availability was evenly distributed between both procedures, any bias that may result from data incompleteness is likely very small. Fourth, we were not able to compare patients with identical BPL and AL lengths as these lengths were very specific in the raRYGB group. This was anticipated by making a pre-selection and only considering patients with similar limb lengths for analysis, thereby excluding patients with, e.g., BPL lengths longer than 70 cm. Lastly, the pouch lengths were not documented, while it has been hypothesized that it might affect weight loss results [41].

Strengths of the current study include that it was one of the first to evaluate the incidence of RWG, which has not extensively been investigated before. Furthermore, to our knowledge, the current study was the first to directly compare raRYGB and RYGB on long-term outcomes with prospectively collected data from a national quality registry. By using real-world data, the results are generalizable to all patients, and propensity score matching enabled us to decrease any confounding by indication bias. Therefore, the current results can be considered high-quality evidence.

### Future Perspectives

Future research should focus on investigating through which action mechanisms ring augmentation results in enhanced weight loss and less RWG. Additionally, exploring the combination of a longer BPL with raRYGB and the effect on obesity complication improvement would be of interest [23, 26]. Finally, potential differences in patient-reported outcomes should be analyzed to ensure that the ring has no negative impact on quality of life.

## Conclusion

Augmentation of an RYGB with the MiniMIZER gastric ring enhanced weight loss over 5 years and reduced the incidence and severity of recurrent weight gain. The additional risk of ring-related complications might be a fair trade-off to the number of patients who will be withheld the need for secondary surgery for suboptimal clinical response. Therefore, based on the current results, performing raRYGB can be a potent and safe option for standard practice.

## Supplementary Information

Below is the link to the electronic supplementary material.

Supplementary file1 (DOCX 15 KB)

## Data Availability

No datasets were generated or analysed during the current study.
