# Correction: Ring Augmentation of the Roux-en-Y Gastric Bypass: A Propensity Score Matched Analysis of 5-Year Follow-Up Results

**DOI:** 10.1007/s11695-025-07734-7

**Published:** 2025-02-13

**Authors:** Marijn T. F. Jense, Floris F. E. Bruinsma, Simon W. Nienhuijs, Ronald S. L. Liem, Perla J. Marang-van de Mheen, Jan Willem M. Greve, Evert-Jan G. Boerma

**Affiliations:** 1 Zuyderland Medisch Centrum, Sittard, Netherlands; 2 Maastricht University, Maastricht, Netherlands; 3 Dutch Institute for Clinical Auditing, Leiden, Netherlands; 4 Catharina Ziekenhuis, Eindhoven, Netherlands; 5 Groene Hart Ziekenhuis, Gouda, Netherlands; 6 Nederlandse Obesitas Kliniek, Zeist, Netherlands; 7 Delft University of Technology, Delft, Netherlands

**Correction: Obesity Surgery**

10.1007/s11695-025-07706-x

For author Perla J. Marang-van de Mheen, the name tagging in the XML is incorrect. The author given name should be “Perla J.”, and the author family name should be “Marang-van de Mheen”.

