# Supplementary material for: Ring Augmentation of the Roux-en-Y Gastric Bypass: A Propensity Score Matched Analysis of 5-Year Follow-Up Results
Source: Obes Surg. 2025 Jan 30;35(3):884–93. doi: 10.1007/s11695-025-07706-x (PMC11906517; doi:10.1007/s11695-025-07706-x)
Supplement: Supplementary file 1 — Supplementary file1 (DOCX 15 KB) [file 11695_2025_7706_MOESM1_ESM.docx]

Supplementary Information

Supplementary table 1. Complementary weight-related outcomes five years after surgery for RYGB vs. raRYGB

|  | **RYGB** | **raRYGB** |
| --- | --- | --- |
|  | **(N = 296)** | **(N = 296)** |
| **Continuous outcomes** |  |  |
| %TWL (mean, SD) | 28.0 (9.7) | 31.5 (8.8) |
| %EWL (mean, SD) | 70.8 (25.1) | 79.9 (23.5) |
| ΔBMI (mean, SD) | 12.0 (4.6) | 13.6 (4.3) |
| **Dichotomous outcomes** |  |  |
| ≥20 %TWL (n, %) | 236 (79.7) | 263 (88.9) |
| ≥25 %TWL (n, %) | 180 (60.8) | 234 (79.1) |
| ≥ 50% EWL (n, %) | 238 (80.4) | 265 (89.5) |

RYGB = Roux-en-Y gastric bypass, raRYGB = ring augmented Roux-en-Y gastric bypass, N = number of patients, %TWL = percentage total weight loss, %EWL = percentage excess weight loss, ΔBMI = absolute change in body mass index (kg/m^2^), SD = standard deviation.

Supplementary table 2. Availability of obesity complication status at 5 years, calculated over the number of patients who had the obesity complication preoperatively.

|  | **N** | **Registered obesity complication status at 5 years (%)** | |
| --- | --- | --- | --- |
|  |  | RYGB | raRYGB |
| Diabetes mellitus | *115* | 87.5 | 79.9 |
| Hypertension | *211* | 89.0 | 77.5 |
| Dyslipidemia | *120* | 70.5 | 81.4 |
| OSAS | *102* | 72.0 | 78.8 |
| GERD | *96* | 43.8 | 31.3 |
| Musculoskeletal pain | *377* | 61.6 | 69.5 |

RYGB = regular Roux-en-Y gastric bypass, raRYGB = ring augmented Roux-en-Y gastric bypass, N = number of patients with the obesity complication at baseline, OSAS = obstructive sleep apnea syndrome, GERD = gastro-esophageal reflux disease.
